# The rhizosphere microbiome improves the adaptive capabilities of plants under high soil cadmium conditions

**DOI:** 10.3389/fpls.2022.914103

**Published:** 2022-10-06

**Authors:** Wenjun Fan, Jinmei Deng, Li Shao, Shiming Jiang, Tangfu Xiao, Weimin Sun, Enzong Xiao

**Affiliations:** ^1^Key Laboratory for Water Quality and Conservation of the Pearl River Delta, Ministry of Education, School of Environmental Science and Engineering, Guangzhou University, Guangzhou, China; ^2^State Key Laboratory of Geohazard Prevention and Geoenvironment Protection, Chengdu University of Technology, Chengdu, China; ^3^National-Regional Joint Engineering Research Center for Soil Pollution Control and Remediation in South China, Guangdong Key Laboratory of Integrated Agro-environmental Pollution Control and Management, Institute of Eco-environmental and Soil Sciences, Guangdong Academy of Sciences, Guangzhou, China; ^4^School of Environment, Henan Normal University, Xinxiang, China; ^5^Key Laboratory of Yellow River and Huai River Water Environment and Pollution Control, Ministry of Education, Xinxiang, China

**Keywords:** rhizosphere microbiome, cadmium, *Sedum alfredii*, assemblage, fitness

## Abstract

Cadmium (Cd) contamination of agricultural soils poses a potential public health issue for humans. Phytoremediation-based accumulating plants are an effective and sustainable technology for Cadmium remediation of contaminated agricultural soil. The rhizosphere microbiome can promote the growth and Cadmium accumulation in hyperaccumulators, but its taxonomic and functional traits remain elusive. The present study used two ecotypes of *Sedum alfredii*, an accumulating ecotype (AE) and a non-accumulating ecotype (NAE), as model plants to investigate the rhizosphere microbiome assemblages and influence on plant growth under high cadmium conditions. Our results showed that distinct root microbiomes assembled in association with both ecotypes of *S. alfredii* and that the assemblages were based largely on the lifestyles of the two ecotypes. In addition, we demonstrated that the functions of the microbes inhabiting the rhizosphere soils were closely associated with root-microbe interactions in both ecotypes of *S. alfredii*. Importantly, our results also demonstrated that the rhizosphere microbiome assembled in the AE rhizosphere soils contributed to plant growth and cadmium uptake under high cadmium conditions through functions such as nitrogen fixation, phosphorus solubilization, indole acetic acid (IAA) synthesis, and siderophore metabolism. However, this phenomenon was not clearly observed in the NAE. Our results suggest that the rhizosphere microbiome plays important roles in biogeochemical nutrient and metal cycling that can contribute to host plant fitness.

## Introduction

Cadmium (Cd) contamination of agricultural soils has dramatically increased worldwide, and the accumulation of Cd in food crops poses a potential public health issue for humans (Zhao et al., 2015; Yue et al., 2016). Phytoremediation-based accumulating plants are an effective and sustainable technology for Cd remediation of contaminated agriculture soil. Generally, Cd accumulation in non-accumulating plants has adverse impacts on their physiological processes, including photosynthesis, respiration, nutrient and water uptake (Khan et al., 2017; Manzoor et al., 2019). In contrast, accumulating plants have evolved effective strategies to grow well under high soil Cd levels (Zhuang et al., 2007; Li et al., 2012), allowing phytoremediation of Cd-contaminated soils. Therefore, these two types of plants exhibit different adaptations in soils under elevated Cd levels.

Existing studies have revealed that roots of certain plants can recruit microbes from surrounding bulk soil to relieve environmental stress, including *Arabidopsis* (Bulgarelli et al., 2012), rice (Edwards et al., 2015), soybean (Mendes et al., 2014), corn (Walters et al., 2018), barley (Bulgarelli et al., 2015), and wheat (Donn et al., 2015). Given that accumulating ecotype (AE) and non-accumulating ecotype (NAE) plants exhibit different adaptive capacities under high Cd levels, it is reasonable to propose that the composition and function of the assembled rhizosphere microbiome of these types of plants may differ under elevated Cd levels. However, it is not clear whether these differences cause soil microbial assemblages to change their adaptive strategies under Cd stress. Answering this question is important for understanding how rhizosphere microbial assemblages contribute to the phytoremediation efficiency under high Cd levels.

The accumulating ecotype (AE) of *S. alfredii* is reported to tolerate and accumulate high levels of Cd (Li et al., 2012). Currently, the AE of *S. alfredii* is widely used for the practical remediation of Cd-contaminated soils in China (Zhuang et al., 2007). Numerous studies have shown that AEs have evolved effective strategies to transfer Cd from soils into plants and to promote plant growth under high soil Cd levels (Zhuang et al., 2007; Li et al., 2012). In contrast, a non-accumulating ecotype (NAE) of *S. alfredii*, an analog, does not grow well under soil Cd stress (Yang et al., 2017). Recently, several studies targeting the rhizosphere microbiome have shown that many rhizosphere microbes from the AE of *S. alfredii* can be characterized as Cd resistant and improve the efficiency of Cd uptake in soils with high Cd contents (Hou et al., 2018). Furthermore, other studies have demonstrated that the rhizosphere microbiome can facilitate growth of the AE of *S. alfredii* through its involvement in indole acetic acid (IAA), aminocyclopropane carboxylic acid (ACC), and nutrient metabolism under high-Cd conditions (Chen Y. et al., 2020). These studies have shown that the rhizosphere microbiome may play an important role in coordinating Cd uptake and growth in *S. alfredii* in soils under Cd stress. However, most of these studies focused only on the taxonomic composition of the rhizosphere microbiome associated with the AE of *S. alfredii*; few studies have systematically investigated the rhizosphere microbial compositions associated with both the AE and the NAE of *S. alfredii*. It is still not clear whether the composition of the rhizosphere microbiome contributes to the difference in adaptability between the two *S. alfredii* ecotypes at high Cd levels. Hence, the results of this study provide detailed information on the taxonomic and functional traits of their respective rhizosphere microbiomes to obtain new knowledge about the functional composition of these rhizosphere microbiomes and their roles in the growth of both ecotypes of *S. alfredii*.

This paper investigates the composition and influence of the rhizosphere microbiota of two ecotypes of *S. alfredii* and their effects on plant growth. To do this, amplicon sequencing was performed to study the taxonomic compositions of rhizosphere and associated bulk soil samples in the two ecotypes of *S. alfredii*. In addition, we selected a subset of samples and performed shotgun metagenomic sequencing to determine the microbial functional compositions of the rhizosphere and associated bulk soil samples in the two ecotypes of *S. alfredii*. The results of this study will provide some important insights into the ecological role of the rhizosphere microbiomes in the review of plant growth under environmental stress.

## Materials and methods

### Environmental setup

Two ecotypes of *S. alfredii*, an AE and a NAE, were used in this study. The AE was obtained from a *S. alfredii* planting base in Zhuzhou (Hunan Province, China), and the NAE was collected from a tea garden in Quzhou (Zhejiang Province, China). The soils used for the pot experiments were collected from the 0 to 20 cm surface layer soils of an abandoned vegetable field located at Guangzhou University (Guangzhou, China). Prior to planting, the collected soils were air-dried, crushed, and sieved. The physicochemical parameters of this soil were determined, including soil pH (averaged 7.07), Cd content (averaged 0.24 mg/kg), total organic carbon (TOC, averaged 2.89%), and total nitrogen (TN, averaged 0.27%). For both ecotypes, the roots were first cut and then grown hydroponically for 2 weeks at 23°C–28°C and a humidity of 70% until they produced hairy roots. A cold-white fluorescent lamp was used to provide light (photon flux, 350 μmol m^−2^ s^−1^; photoperiod, 14 h). Vigorously aerated Hoagland-Arnon nutrient media (0.2 strength; Hoagland and Arnon, 1950) were applied to the plants and replenished twice a week to maintain plant growth. After 2 weeks of acclimatization, the plants were transferred into soils containing 0 (denoted as the control), 100 (denoted as Cd_100), or 500 mg/kg Cd (denoted as Cd_500; CdCl_2_, Sigma, St. Louis, MO). Each treatment included 4 replicates for quality control purposes. Deionized water was supplied to the pots every 3 days to maintain a relative soil moisture level ranging from 50% to 70%. The plants, rhizosphere soil and bulk soil were harvested after 90 days of Cd treatment. The samples were divided into two parts at different storage temperatures. The samples used in the chemical analysis were stored at 4°C. The samples used in the microbial analysis were stored at −80°C.

### Sample collection

The rhizosphere and bulk soil were collected. The rhizosphere soil is composed of ∼1 mm of soil tightly adhering to the root surface that is not easily shaken from the root. We removed the plant from each pot and then manually shook the excess soil from the roots to leave approximately 1 mm of soil attached to the roots. These roots were placed in a sterile flask with 50 ml of sterile phosphate-buffered saline (PBS) solution and then stirred vigorously with sterile forceps to separate the roots and soil (Edwards et al., 2015). The soil that was removed from the roots was poured into a 50 ml Falcon tube and stored as the rhizosphere soil at 4°C until DNA extraction.

### Chemical analyses

Prior to chemical analysis, the samples were freeze-dried (−80°C), thoroughly ground, and sieved (<0.075 mm). Soil pH was determined using a pH meter (HACH, Loveland, United States) after mixing 10 g of soil with 25 ml of MQ water in a 50 ml conical flask. The total nitrogen (TN) and total organic carbon (TOC) contents were measured using an elemental analyzer (Vario MACRO cube, Elementar, Hanau, Germany). The sieved samples were then completely digested using concentrated hydrofluoric acid and nitric acid (1:5, v/v) before elemental analysis (Xiao et al., 2021b). After digestion, the contents of Cd were measured using inductively coupled plasma mass spectrometry (ICP-MS, Agilent, 7700x, California, United States; Liu et al., 2020; Wang et al., 2021). To improve the reliability of ICP-MS, certified references (SLRS-5 and GBW07310) were used during the measurements following our previous studies (Xiao et al., 2021b).

### Analyses of microbial communities

For the total genomic DNA extraction, 0.25 g of soil was extracted using the FastDNA^®^ Spin Kit (MP bio, Santa Ana, United States) following the manufacturer’s protocol. Prior to sequencing, the primer pair 515f/907r was used to amplify the V4-V5 regions of the 16S rRNA amplicons (Kuczynski et al., 2012). The PCR amplied products were purified using a DNA gel extraction kit (Axygen, China) and were quantified using a Qubit dsDNA HS Assay Kit (Life Technologies, Carlsbad, CA, United States). The 16S rRNA amplicons were then sequenced on the Illumina MiSeq platform at Shanghai Majorbio Biopharm Technology Company (Shanghai, China). The paired-end reads were merged and assigned to each sample following the proposed protocol (Magoč and Salzberg, 2011). We then filtered the raw reads using split_libraries_fastq.py in QIIME (V1.7.0; Bokulich et al., 2013) and removed chimeric sequences based on the GOLD database by using UCHIME^1^ (Haas et al., 2011). We obtained operational taxonomic units (OTUs) at a 97% similarity threshold by using UPARSE and then obtained taxonomic information for each sample using the RDP classifier^2^ and the Greengenes database^3^ (Wang et al., 2007).

### Shotgun metagenomic sequencing analysis

In this study, we selected 12 samples for shotgun metagenomic sequencing analysis on the Illumina MiSeq platform at Shanghai Majorbio Biopharm Technology Company (Shanghai, China). The clean data were obtained by removing low-quality and ambiguous sequence reads by using Readfq (V8, https://github.com/cjfields/readfq). Then, the clean data were assembled by using MEGAHIT software (v1.0.4-beta; Mende et al., 2012; Nielsen et al., 2014). PE reads were obtained by comparing the clean data from all samples with each scaffold by using Bowtie2.2.4 software. The unigenes were blast against the KEGG database (Version 201,609, *E*-value ≤ 1e^−5^, http://www.kegg.jp/kegg/) by using DIAMOND software (V0.9.9; Minoru et al., 2014). The taxonomy profiles of unigenes were blast against the NR database (Version nr_v20200604, *E*-value ≤ 1e^−5^) using DIAMOND software (V0.9.9, https://github.com/bbuchfink/diamond/) with default settings (Hu et al., 2017). The datasets of the 16S rRNA and metagenomic sequences presented in this study can be found in NCBI under accession numbers SRP367081 and SRP366399, respectively.

### Statistical analysis

Student’s *t*-test was used to determine differences in the fresh weight, dry weight, root length, plant Cd and soil Cd between the AE and NAE *S. alfredii* ecotypes. The Shapiro–Wilk test was used to verify the normal distribution of the data, followed by Student’s *t*-test using the t.test() function; *p*-values <0.05 were considered statistically significant in this study (indicated by an asterisk in the figure). We analyzed the enrichment of OTUs and genera between the two *S. alfredii* ecotypes by using the “tidyverse” and “DESeq2” packages in R software (Love et al., 2014) The significantly enriched and depleted OTUs in the volcanic map were displayed at the phylum level using a stacked histogram. The differences in metabolic potential and functions were visualized between the two *S. alfredii* ecotypes using STAMP, which provided effect sizes and confidence intervals.

## Results

### Growth performance of the two *Sedum alfredii* ecotypes

**Figure 1:**
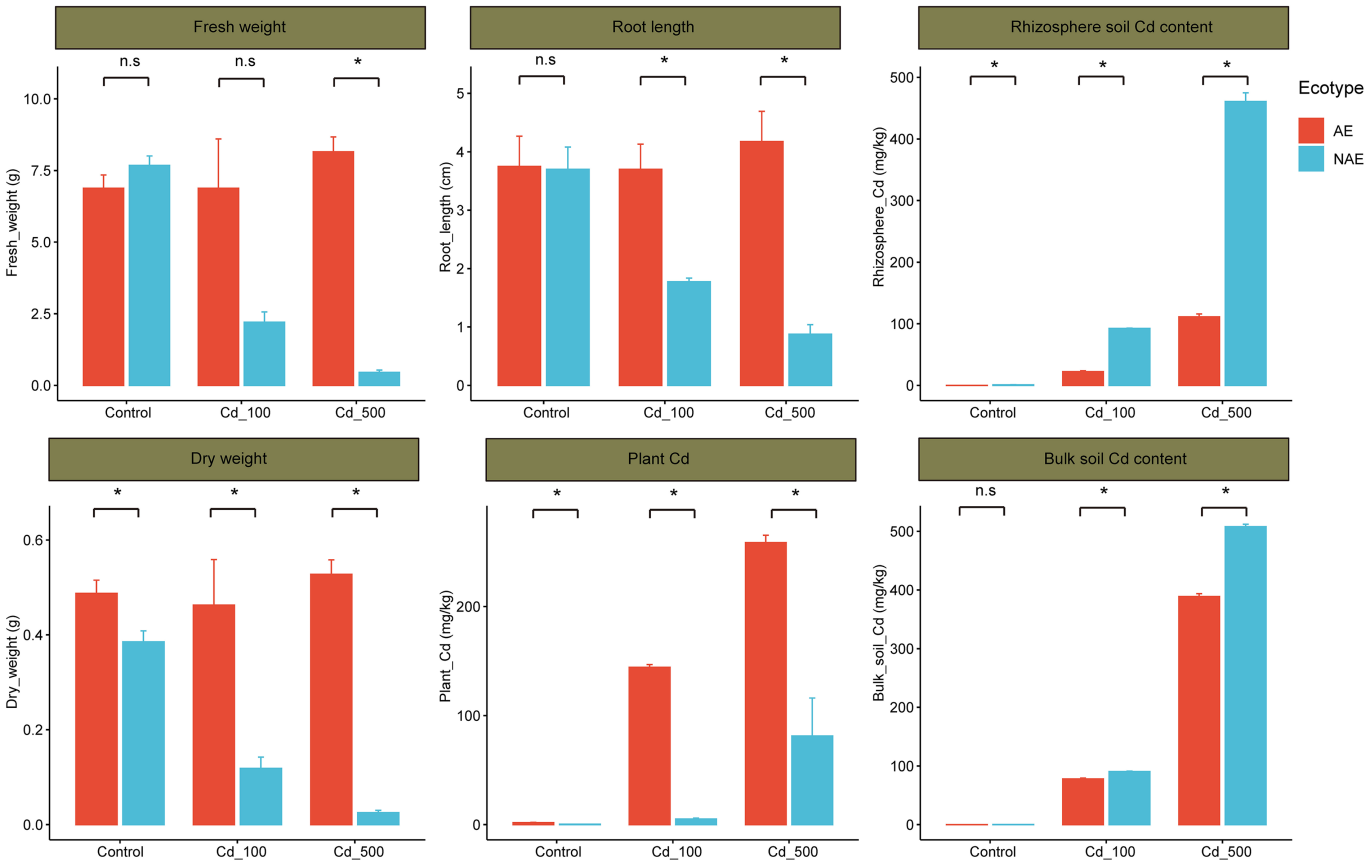
Distribution of the plant growth performance (fresh weight, dry weight and root length, plant Cd contents, and rhizosphere soil Cd contents and bulk soil Cd contents) between AE and NAE ecotypes of *Sedum alfredii* in control, Cd_100, and Cd_500 groups. *p < 0.05.

The fresh and dry weights of the AE and the NAE of *S. alfredii* exhibited opposite trends as the Cd content increased from 0 to 500 mg/kg. For example, the fresh and dry weight of the AE of *S. alfredii* were higher in Cd_500 group (fresh weight: 8.16 g, dry weight: 0.53 g) than that in Cd_100 group (fresh weight: 6.88 g, dry weight: 0.46 g) and control (fresh weight: 6.88 g, dry weight: 0.49 g). In contrast, the fresh and dry weight of the NAE of *S. alfredii* were lower in Cd_500 group (fresh weight: 0.45 g, dry weight: 0.03 g) than that in Cd_100 group (fresh weight: 2.21 g, dry weight: 0.19 g) and control group (fresh weight: 7.68 g, dry weight: 0.39 g). Similarly, the root length of the AE of *S. alfredii* were relative higher in Cd_500 group (4.2 cm) than that in Cd_100 group (3.7 cm) and control group (3.8 cm). In contrast, the root length of the NAE of *S. alfredii* were lower in Cd_500 group (0.9 cm) than that in Cd_100 group (1.8 cm) and control group (3.7 cm). Notably, fresh weight, dry weight and root length were significant higher in the AE of *S. alfredii* than in the NAE of *S. alfredii* in the Cd_100 and Cd_500 treatments, but there were no significant differences in the fresh and dry weights and root length between the ecotypes in the control treatment (Figure 1, *t*-test, *p* < 0.05). As shown in Supplementary Figure S1, the average rhizosphere soil Cd content in the AE of *S. alfredii* was lower than that in the bulk soils with increasing Cd levels (*t*-test, *p* < 0.05). For example, for the AE of *S. alfredii*, the average rhizosphere soil Cd level was 22.05 mg/kg, whereas the average bulk soil Cd level was 97.54 mg/kg in the Cd_100 treatment; in the Cd_500 treatment, the average rhizosphere soil Cd level was 111.20 mg/kg, whereas the average bulk soil Cd level was 498.32 mg/kg. In contrast, the average soil Cd level was not obviously different between the rhizosphere and bulk soil across all Cd treatments in the NAE of *S. alfredii* (*t*-test, *p* < 0.05). For example, for the NAE of *S. alfredii*, the average rhizosphere soil Cd level was 92.4 mg/kg, and the average bulk soil Cd level was 90.43 mg/kg in the Cd_100 treatment; in the Cd_500 treatment, the average rhizosphere soil Cd level was 460.82 mg/kg, whereas the average bulk soil Cd level was 507.92 mg/kg. Notably, the average whole-plant Cd content in the AE of *S. alfredii* was significantly higher than that in the NAE of *S. alfredii* across all Cd treatments (Figure 1, *t*-test, *p* < 0.05). For example, the average whole-plant Cd content for the AE of *S. alfredii* was 144.37 mg/kg, whereas the average whole-plant Cd content for the NAE of *S. alfredii* was 5.2 mg/kg in the Cd_100 treatment; in the Cd_500 treatment, the average whole-plant Cd content for the AE of *S. alfredii* was 258.75 mg/kg, whereas the average whole-plant Cd content for the NAE of *S. alfredii* was 81.23 mg/kg.

### Results of 16S rRNA amplicon sequencing

Approximately 5.48 M reads (113,663 reads per sample) and 6,606 OTUs were generated for the bacterial community composition analysis (Supplementary Table S1). As shown in Supplementary Figure S2, the microbial community composition at the phylum level consisted mainly of *Actinobacteria* (28%), *Proteobacteria* (27%), *Acidobacteria* (15%), *Chloroflexi* (11%), *Firmicutes* (6%), and *Bacteroidetes* (3%). Notably, the relative abundances of the dominant phyla were not significantly different between the rhizosphere and the associated bulk soil samples in either *S. alfredii* ecotype. For this reason, we obtained detailed taxonomic information to characterize the microbial compositions of the rhizosphere and bulk soil samples.

**Figure 2:**
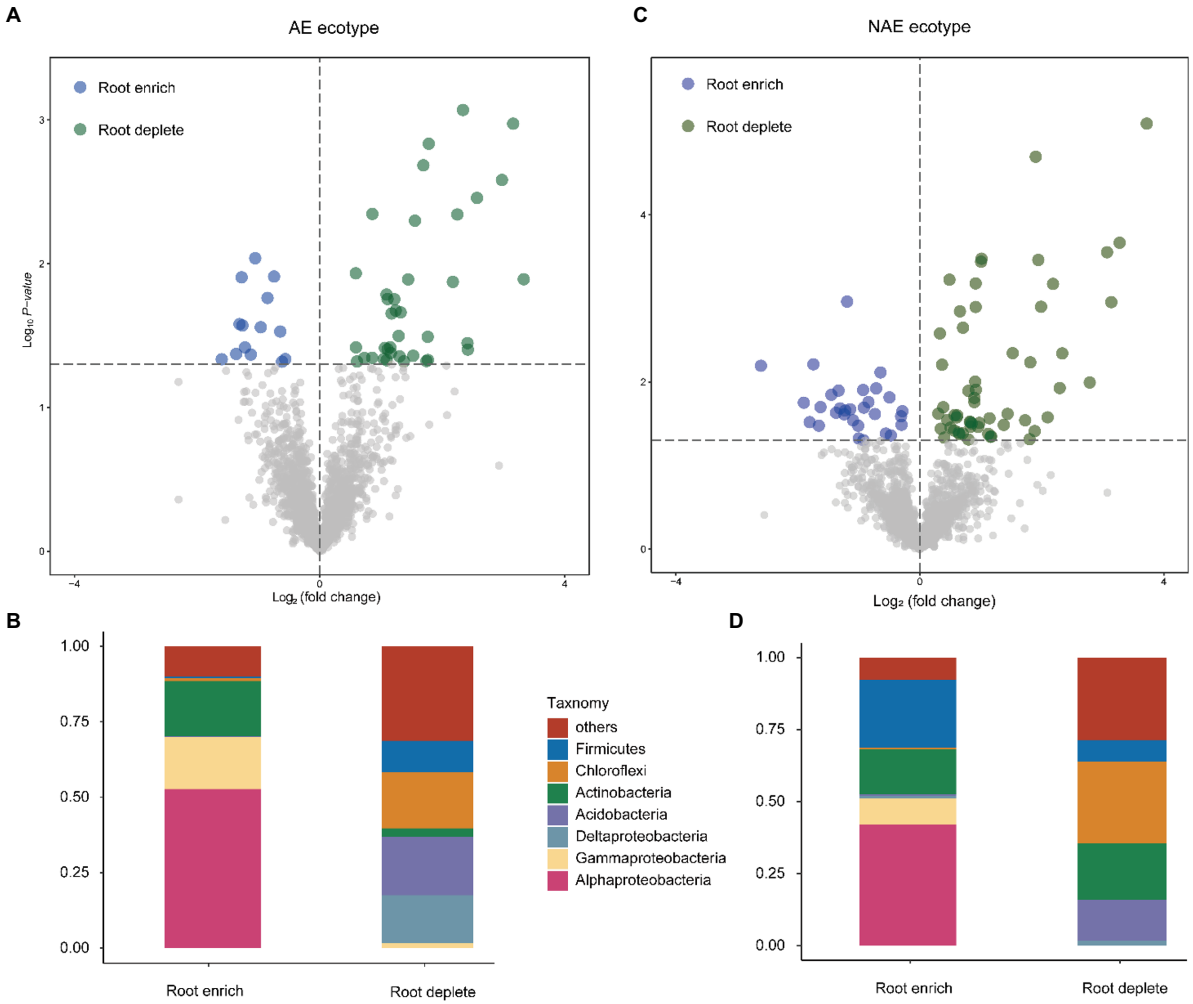
Distribution pattern of dominant OTUs and the taxonomy composition (phylum level) between root enrich and deplete in AE *S. alfredii* ecotype **(A,B)** and NAE *S. alfredii* ecotype **(C,D)**; “others” refers to other low abundant phyla.

**Figure 3:**
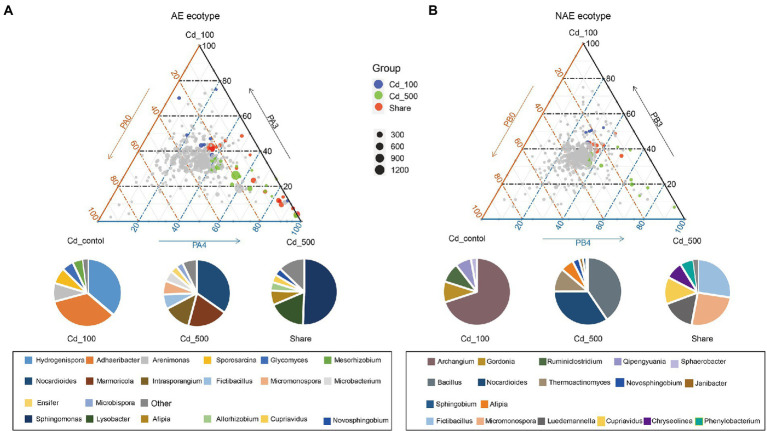
Distribution pattern of dominant genera and the taxonomy composition (genus level) across Cd_control, Cd_100, and Cd_500 for **(A)** AE *S. alfredii* ecotype and **(B)** NAE *S. alfredii* ecotype.

We assessed the distribution of the OTUs between the rhizosphere and the associated bulk soils using linear model analysis for both *S. alfredii* ecotypes (Figure 2; Supplementary file Dataset S1). The results show that the OTUs were grouped into two distinct subcommunities in the rhizosphere and bulk soil in both the AE and the NAE of *S. alfredii*. After taxonomic assignment, we found that in the AE of *S. alfredii*, the OTUs enriched in the rhizosphere soils consisted primarily of *Alphaproteobacteria*, *Gammaproteobacteria*, and *Deltaproteobacteria*, whereas the OTUs enriched in the bulk soils consisted of *Firmicutes*, *Chloroflexi*, *Actinobacteria*, and *Acidobacteria*. For the NAE, the OTUs enriched in the rhizosphere soils consisted primarily of *Alphaproteobacteria* and *Gammaproteobacteria*, whereas the OTUs enriched in the bulk soils were *Chloroflexi*, *Actinobacteria*, and *Acidobacteria*. To obtain detailed taxonomic information for the rhizosphere soils across all Cd treatments in both *S. alfredii* ecotypes (Supplementary file Dataset S2), we compared the enrichment of bacterial genera in the Cd_100 and Cd_500 treatments with that in the control treatment (Figure 3). For the AE of *S. alfredii*, the genera enriched in the Cd_100 and Cd_500 soils were *Sphingomonas*, *Lysobacter*, *Afipia*, *Hydrogenispora*, *Luedemannella*, *Adhaeribacter*, *Allorhizobium-Neorhizobium-Pararhizobium-Rhizobium*, *Cupriavidus*, *Microbacterium*, *Novosphingobium*, *Massilia*, *Janibacter*, *Sphingobium*, *Nakamurella*, *Paenarthrobacter*, *Symbiobacterium*, *Achromobacter*, *Azospirillum*, and *Caulobacter*. For the NAE, the genera enriched in the Cd_100 and Cd_500 soils were *Bacillus*, *Nocardioides*, *Thermoactinomyces*, *Fictibacillus*, *Micromonospora*, *Luedemannella*, *Cupriavidus*, *Chryseolinea*, *Phenylobacterium*, *Gordonia*, *Ruminiclostridium*, *Qipengyuania*, *Novibacillus*, *Thermomonas*, *Salinispora*, *Sphaerobacter*, *Pseudobacteroides*, and *Demequina*.

### Results of shotgun metagenomic sequencing analysis

**Figure 4:**
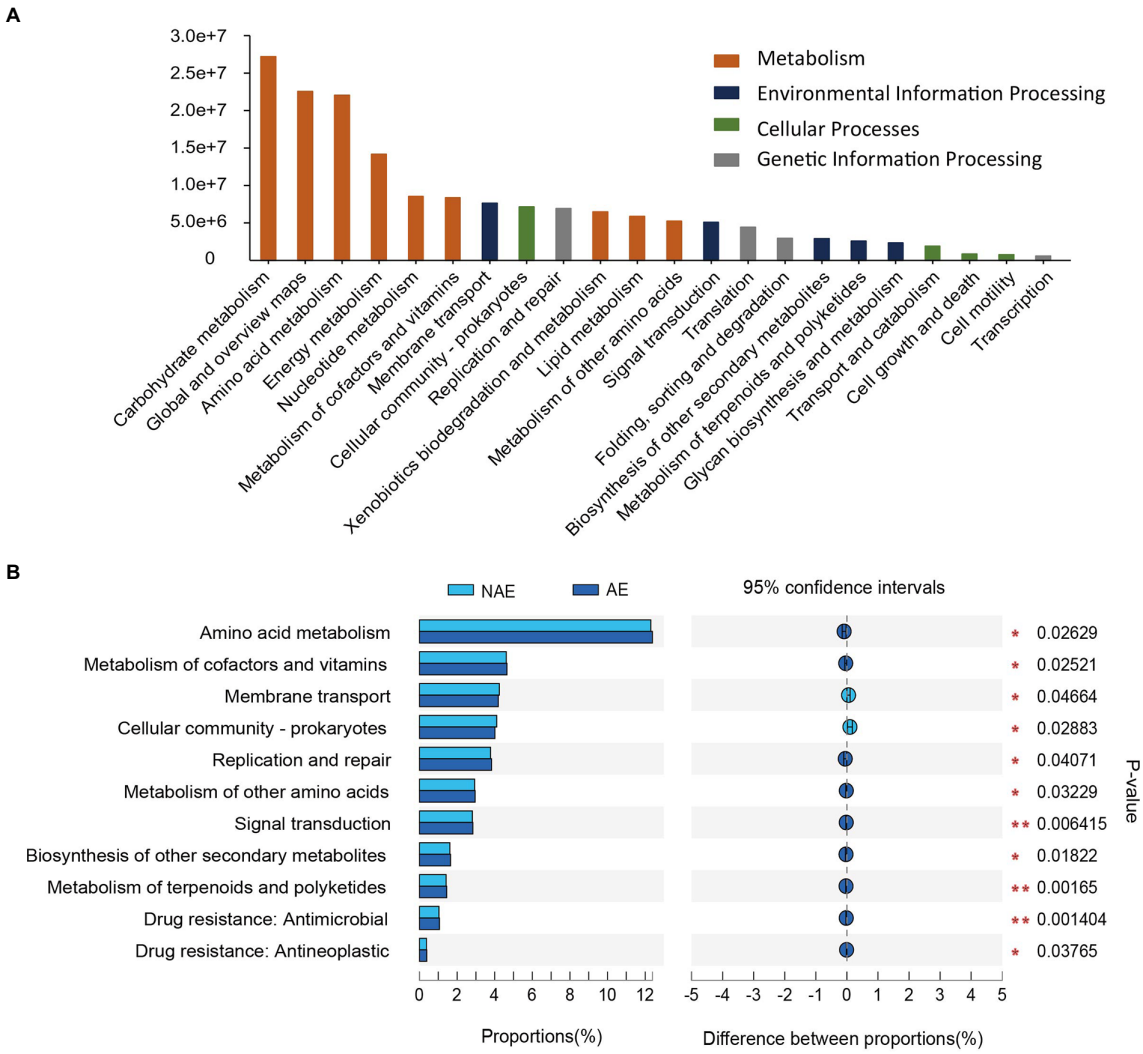
**(A)** The metabolic potential pathways of the two ecotypes of *S. alfredii* rhizosphere microbiome and **(B)** and their relative abundance in rhizosphere soils between AE and NAE *S. alfredii* ecotype.

**Figure 5:**
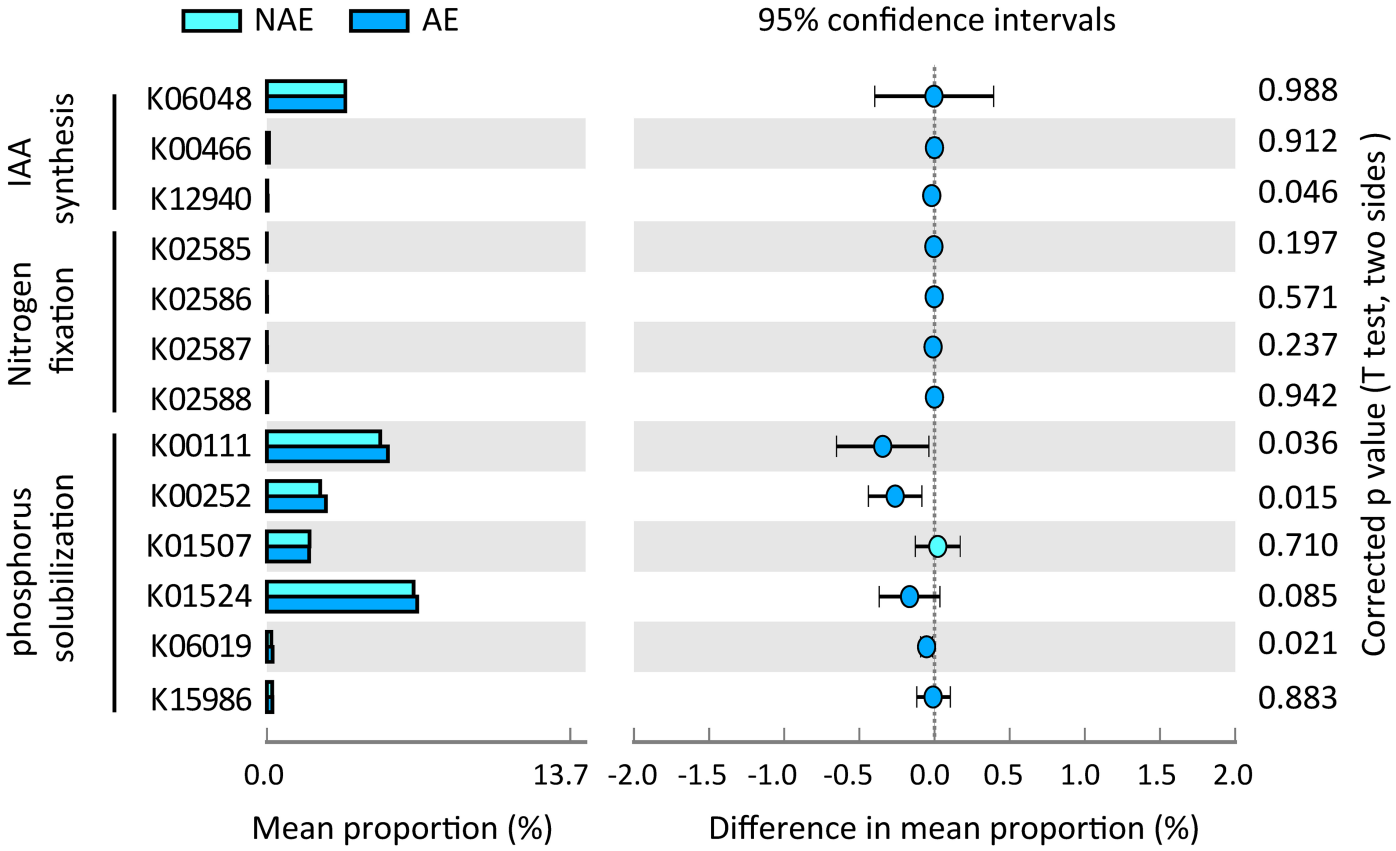
Distribution of microbial functions involving into IAA synthase, Nitrogen fixation, and P solubilization in rhizosphere soils between AE and NAE ecotypes of *S. alfredii*.

**Figure 6:**
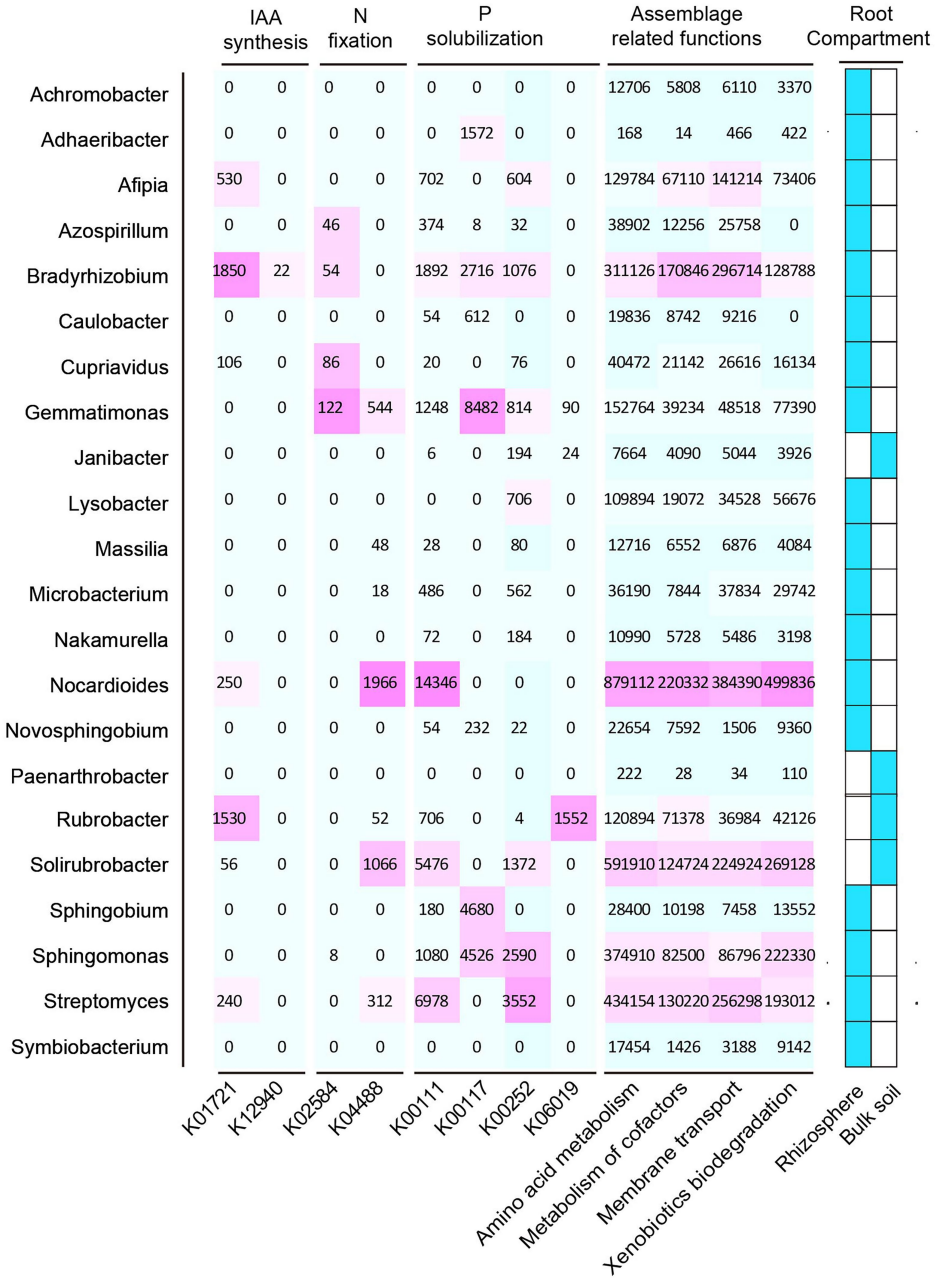
Distribution of microbial functions of IAA synthase, Nitrogen fixation, and P solubilization and root assemblage related functions in their hosts.

In this study, we selected 12 samples for shotgun metagenomic sequencing to capture the microbial functional traits of the rhizosphere and bulk soils under both ecotypes of *S. alfredii*. We obtained a total of 7,511 KEGG orthologs (KOs) in all samples. As shown in Figure 4A, these KOs were associated with 4 *level 1* and 22 *level 2* KEGG pathways. Among the *level 2* KEGG pathways, the metabolism pathway was the most abundant in all samples and consisted mainly of amino acid metabolism, carbohydrate metabolism, and energy metabolism. Notably, 11 of the 22 *level 2* KEGG pathways were significantly different between the AE and NAE rhizosphere soils (Figure 4B). To obtain detailed information about the microbial functions involved in plant growth between the *S. alfredii* ecotypes in the rhizosphere soils, we compared the significantly enriched microbial functions between the AE and NAE *S. alfredii* ecotypes. For the AE of *S. alfredii*, the enriched KOs were IAA synthesis (K12940), nitrogen fixation (K02585, K02586, K02587, and K02588), P solubilization (K00252, K00111, and K06019; Figure 5). Furthermore, we found that the KOs involved in IAA synthesis (K01721 and K12940), nitrogen fixation (K02584 and K04488), and P solubilization (K00117, K00111, K00252, and K06019) were affiliated with *Bradyrhizobium*, *Gemmatimonas*, Nocardioides, and *Sphingomonas* (genus level; Figure 6).

## Discussion

Elevated soil Cd contents generally adversely impact plant growth (Li et al., 2012). However, the current study showed that the two ecotypes of *S. alfredii* exhibited differences in Cd tolerance. Compared with the control, the growth of the NAE *S. alfredii* grown in Cd-treated soil was significantly decreased. In contrast, no obvious growth inhibition or symptoms of phytotoxicity were observed for the AE *S. alfredii*. This result is consistent with those of prior studies suggesting that a high level of metals could differentially impact the growth performance of these two ecotypes (Zhuang et al., 2007; Li et al., 2012; Hou et al., 2018). Furthermore, we identified that the two ecotypes of *S. alfredii* had differences in Cd accumulation. The average plant Cd content for the AE plants was significantly higher than that for the NAE of *S. alfredii* across all Cd treatments (Figure 1). Furthermore, the Cd that accumulated in the plants corresponded to the Cd depletion in the rhizosphere soil (Figure 1). These results suggest that the AE *S. alfredii* accumulates more Cd from the rhizosphere soil than the NAE of *S. alfredii*. Similarly, Li et al. (2012) demonstrated that the AE *S. alfredii* facilitates Cd uptake from rhizosphere soils into plants. Because of its relatively high capacity for extracting Cd from soil, AE *S. alfredii* could decrease total Cd levels in its rhizosphere soil and therefore could be useful in the phytoremediation of Cd-polluted soils (Zhuang et al., 2007). Recent evidence shows that the potential of the AE *S. alfredii* for extracting Cd relies not only on its physiological process but also on processes belowground that facilitate root uptake and tolerance of Cd (Hou et al., 2018; Chen J. et al., 2020). Therefore, we applied a high-throughput sequencing approach to gain insight into the soil–plant-microbe interactions that may influence Cd accumulation in the two ecotypes of *S. alfredii*.

Previous study has demonstrated that plant roots could recruit microbes from the surrounding bulk soils to improve the stress tolerance of their host plants (Bulgarelli et al., 2012). The current results showed that the OTUs could be classified into two groups, those in the rhizosphere and those in the bulk soils, in both the AE and the NAE of *S. alfredii* (Figure 2). Importantly, the rhizosphere soil-enriched OTUs were mainly identified as fast-growing bacteria that use various carbon sources, whereas the bulk soil-enriched OTUs were mainly identified as slow-growing bacteria that use only a few carbon sources (Leff et al., 2015; Xiao et al., 2021a). This result was agreed to prior work that plant roots recruit microorganisms from the associated bulk soils on the basis of their lifestyles (Leff et al., 2015). Recent evidence suggests that the evolution of microbial functions involved in plant-microbe interactions is linked to the assemblage of the rhizosphere microbiome in rhizosphere soils (Hassani et al., 2018). The current study showed that microbial functions involved in rhizosphere–microbe interactions were detected in rhizosphere soil for both the AE and NAE of *S. alfredii* (Figure 3A). These functions have been reported to be able to regulate diverse adaptation processes, such as nutrient competition and environmental stress (Berendsen et al., 2018), which contribute to microbial assembly across the soil-root interface. These results revealed that microbial assemblage occurs across the root interface and is based on the lifestyles of the microbes for both the AE and NAE of *S. alfredii*.

The current study demonstrated that the assembled microorganisms in the rhizosphere of the AE of *S. alfredii* were those that have been reported to have plant growth-promoting and Cd requisition abilities under high-Cd conditions. For instance, the genera enriched in the rhizosphere of the AE of *S. alfredii*, such as *Achromobacter*, *Sphingobium*, *Novosphingobium*, *Gemmatimonas*, *Cupriavidus*, *Nakamurella*, and *Nocardioides* (Figure 4), could influence plant growth *via* IAA production, nitrogen fixation, and P solubilization and help plants obtain metals from the soil (Videira et al., 2009; Hayat et al., 2013; Rangjaroen et al., 2017). Specifically, prior evidence showed that a member of *Achromobacter* could not only promote plant growth *via* N fixation, IAA production, and P solubilization (Jha and Kumar, 2009) but could also enhance Cd tolerance (Tamariz-Angeles et al., 2021) and As transformation abilities (Corsini et al., 2018). In addition, *Sphingobium* and *Novosphingobium* were identified as P-solubilizing bacteria that have the capacity to solubilize P complexes into bioavailable forms by the process of acidification. Moreover, these two genera were reported to exhibit the capacity to use a wide range of root exudates (Krishnan et al., 2017) and mobile Ni from rhizosphere soils (Chen J. et al., 2020). *Gemmatimonas*, the dominant species in rhizosphere soils of the AE of *S. alfredii*, were reportedly involved in metal mobilization by enhancing microbial nitrogen fixation (Hu et al., 2021). Members of *Cupriavidus* and *Nocardioides* showed plant growth-promoting capacities *via* P and K solubilization and were also reportedly involved in metal resistance and mobilization in rhizosphere soils (Abdulla, 2009). Importantly, these rhizosphere bacteria were not significantly enriched in rhizosphere soils of the NAE of *S. alfredii* under high-Cd conditions. Therefore, our study raises the intriguing possibility that the assembled rhizosphere microbiome plays a beneficial role in plant growth-promotion and may influence Cd requisition, thereby contributing to the discrepancy between the adaptive capabilities of the AE and NAE of *S. alfredii* under high-Cd conditions.

To further examine the relevance of microbial plant growth-promoting functions and Cd uptake, we used shotgun metagenomic sequencing to analyze microbial plant growth-promoting metabolic pathways, including IAA synthesis, nitrogen fixation, and P solubilization. The results showed that a microbial IAA synthesis gene nitrile hydratase (K01721) and an aminobenzoyl glutamate utilization protein (K12940) were significantly enriched in the rhizosphere soils of the AE of *S. alfredii* (Figure 4). This result is reasonable because IAA synthesis in the AE rhizosphere soils could impact root elongation and therefore stimulate growth and protect plants against metal stress (Taghavi et al., 2009). Existing evidence suggested that the accumulation of microbial IAA synthesis genes efficiently mobilized Cd from soils, which promotes Cd requisition in *Brassica napus* (Li et al., 2019). Notably, the current study showed that an IAA synthesis gene is significantly correlated with the Cd content in rhizosphere soils (Figure 5). Importantly, we found that microbial functions involved in IAA synthesis were affiliated with *Bradyrhizobium* (Figure 5), which has been widely identified in Cd-impacted environments and reported to tolerate elevated levels of Cd. A study showed that *Bradyrhizobium* has been widely identified in rhizosphere soils of various plants and has the capacity to mobilize metals *via* IAA synthesis (Liu et al., 2015). Recent evidence has shown that root exudates can regulate the distribution of the microbial functions involved in IAA synthesis (Ma et al., 2011). Therefore, it is reasonable to suggest that the assembled rhizosphere microbiome plays a beneficial role in IAA synthesis and improves the Cd requisition of the AE of *S. alfredii*, thereby contributing to the discrepancy between the adaptive capabilities of the AE and NAE of *S. alfredii* under high-Cd conditions.

Microbial nitrogen fixation converts atmospheric dinitrogen into ammonia, a type of biologically active nitrogen, to support the growth of both microbes and the host plant in nutrient-stressed environments (Tao et al., 2019). Generally, microbial mediated nitrogen fixation can also secrete a variety of organic acids to mobilize metals (Hu et al., 2021). In the current study, the fixK gene, which encodes a nitrogen fixation-regulating protein, was enriched in the AE rhizosphere soils, demonstrating that nitrogen-fixing genes are overrepresented in the AE of *S. alfredii* (Figure 4). This result was reasonable because an elevated level of bioavailable Cd could facilitate the growth of the AE but inhibit the growth of the NAE of *S. alfredii* (Li et al., 2012). Researchers discovered a general consistency between the nitrogen-fixing genes and 16S rRNA genes (Sun et al., 2018). The results showed that the fixK gene sequences were primarily from *Gemmatimonas* and *Nocardioides* at the genus level. *Gemmatimonas* and *Nocardioides* were widely identified in metal-impacted environments and tolerated elevated levels of Cd (Abdulla, 2009; Hu et al., 2021). These facts indicate the potential that elevated Cd levels not only regulate the enrichment of microorganisms with microbial nitrogen fixation functions to alleviate soil N but also mobilize Cd from the rhizosphere soil, which is consistent with previous studies (Chen Y. et al., 2020). Similar to nitrogen fixation genes, genes associated with microbial functions involving P solubilization, including glpA (K00111), gcd (K00117), gcdH (K00252), and ppaX (K06019), were more highly enriched in the AE than that in the NAE rhizosphere soils. Microbial P solubilization converts insoluble phosphates into available P forms by secreting a variety of organic acids to promote host fitness in P-limited environments (Brucker et al., 2020). Importantly, microbial P solubilization was also reported to mobilize Cd from soils by increasing the soil acidity (Chen Y. et al., 2020). The microbial P solubilization gene is widely distributed in microbes in Cd-contaminated environments (Zuniga-Silva et al., 2016). In addition, this gene was affiliated with *Gemmatimonas* and *Sphingomonas* at the genus level, both of which were enriched in the rhizosphere microbiomes of the AE of *S. alfredii*. *Gemmatimonas* and *Sphingomonas* were not only important for P dissolution (Lu et al., 2020) but were also reported to tolerate metals in impacted environments (Hu et al., 2021). These facts suggest that the assembled microbes facilitate microbial-mediated nitrogen fixation and P solubilization and thereby may be involved in Cd uptake by *S. alfredii* roots.

### Contribution

Due to its high capacity for extracting Cd, accumulating ecotype (AE) of *S. alfredii* has been widely applied in the remediation of cadmium-contaminated soils in China. Current evidence suggests that the rhizosphere microbiome can promote the growth and Cd accumulation in AE *S. alfredii*, but its taxonomic and functional traits remain elusive. This study demonstrated that the rhizosphere microbiome assembled in the AE rhizosphere soils contributed to plant growth and Cd uptake under high-Cd conditions through functions such as nitrogen fixation, P solubilization, and indole acetic acid (IAA) synthesis. However, this phenomenon was not clearly observed in the non-accumulating ecotype (NAE) of *S. alfredii*. We believe that our results provide some new insights into the disparate adaptive capabilities of plants under metal-stress conditions and thereby improve our understanding of how the rhizosphere microbiome contributes to the fitness of host plants under environmental stress.

## Data availability statement

The datasets of the 16S rRNA and metagenomic sequences presented in this study can be found in NCBI under accession numbers SRP367081 and SRP366399, respectively.

## Author contributions

EX, TX, and WS contributed to conception and design of the study. WF and JD wrote the first draft of the manuscript. SJ performed the statistical analysis. All authors contributed to manuscript revision, read, and approved the submitted version.

## Funding

This research was funded by the National Natural Science Foundation of China (41830753, 41807127, and U1612442).

## Conflict of interest

The authors declare that the research was conducted in the absence of any commercial or financial relationships that could be construed as a potential conflict of interest.

## Publisher’s note

All claims expressed in this article are solely those of the authors and do not necessarily represent those of their affiliated organizations, or those of the publisher, the editors and the reviewers. Any product that may be evaluated in this article, or claim that may be made by its manufacturer, is not guaranteed or endorsed by the publisher.

## Footnotes

^1^ http://www.drive5.com/usearch/manual/uchime_algo.html

^2^ http://sourceforge.net/projects/rdp-classifier/

^3^ https://greengenes.lbl.gov/Download/
